# Chaperonin containing TCP1 subunit 3 (CCT3) promotes cisplatin resistance of lung adenocarcinoma cells through targeting the Janus kinase 2/signal transducers and activators of transcription 3 (JAK2/STAT3) pathway

**DOI:** 10.1080/21655979.2021.1971030

**Published:** 2021-10-06

**Authors:** Xu Danni, Zeng Jiangzheng, Sun Huamao, Pan Yinglian, Yang Changcheng, Lu Yanda

**Affiliations:** Department of Oncology, The First Affiliated Hospital of Hainan Medical College, Haikou, Hainan, China

**Keywords:** CCT3, cisplatin resistance, non-small cell lung carcinomas, pathway

## Abstract

Cisplatin resistance remains a major obstacle to effective chemotherapies for non-small cell lung cancer (NSCLC). Chaperonin containing TCP1 subunit 3 (CCT3) has been extensively investigated in various cancers, but not in the context of drug resistance. In the present study, we aimed to investigate the role of CCT3 in cisplatin resistance of lung adenocarcinoma (LUAD) cells. By surveying the Gene Expression Profiling Interactive Analysis (GEPIA) website, we found CCT3 expression to be up-regulated in NSCLCs, which correlated with the poor prognosis of LUAD patients. Furthermore, both mRNA and protein levels of CCT3 were upregulated in the cisplatin-resistant A549/DDP cells compared to the cisplatin-sensitive A549 cells. Importantly, upon cisplatin treatment, short hairpin RNA (shRNA)-mediated CCT3 knockdown significantly inhibited the proliferation, invasion and migration of A549/DDP cells, and induced significant G2/M cell cycle arrest and apoptosis in A549/DDP cells. Moreover, CCT3 knockdown significantly weakened the tumorigenicity of the cisplatin-treated A549/DDP cells *in vitro* and *in vivo*. Finally, CCT3 knockdown re-sensitized A549/DDP cells to cisplatin through inhibiting the Janus kinase 2/signal transducers and activators of transcription 3 (JAK2/STAT3) pathway. In conclusion, our results demonstrated that CCT3 could promote cisplatin resistance of LUAD cells via activating the JAK2/STAT3 pathway, indicating that CCT3 may be a novel molecular target for overcoming cisplatin resistance in LUAD patients.

## Introduction

Lung cancer is the most prevalent cancer and the leading cause of cancer death in both sexes combined [1]. There are two main histological types of lung cancer, small cell lung cancer and non-small cell lung cancer (NSCLC), the latter of which accounts for 80–85% of all lung cancer cases [2,3]. Approximately 50% of NSCLC patients are diagnosed at an advanced clinical stage, rendering them ineligible for radical treatment [4]. Therefore, cisplatin (DDP)-based chemotherapy remains a widely used regimen for patients with unresectable NSCLCs [5]. Nevertheless, drug resistance can frequently affect the efficacy of chemotherapy, and consequently result in therapeutic failure and tumor recurrence [6]. Hence, the mechanisms underlying chemotherapy resistance need to be further explored to provide insights into future therapeutic strategies.

Chaperonin containing TCP1 (CCT, also termed TRiC) consists of eight subunits (CCT1–8) and possesses a unique intra- and inter-ring structure [7]. CCT is considered a key regulator involved in the folding and assembly of cytosolic proteins [8]. An important subunit of CCT, CCT3, has been extensively studied in various cancer contexts. For instance, CCT3 overexpression was proposed to predict poor prognosis in patients with hepatocellular carcinoma [9]. Furthermore, the proliferation and colony formation of gastric and breast cancer cells were significantly suppressed upon CCT3 knockdown *in vitro* and *in vivo* [10,11]. In spite of the mounting evidence indicating that CCT3 is critical for tumorigenesis and cancer progression, the potential role of CCT3 in regulating drug resistance has not been investigated.

Signal transducer and activator of transcription 3 (STAT3) is an important downstream signaling molecule of numerous growth factors and cytokines, and participates in various biological processes, such as cell proliferation, differentiation, and survival [12]. STAT3 can be activated by nonreceptor tyrosine kinases such as Janus kinases (JAKs) in a tyrosine phosphorylation dependent-manner [13]. The JAK2/STAT3 pathway has been considered a promising target for chemotherapeutic interference ascribed to its persistent activation in human carcinomas [14]. This pathway is also found to be involved in drug resistance in diverse cancers, such as gemcitabine resistance in pancreatic cancer [15], cisplatin resistance in nasopharyngeal carcinoma [16], and epirubicin resistance in NSCLCs [17].

Interestingly, several recent studies have linked the CCT3 subunit to the JAK/STAT3 pathway. For instance, a bioinformatics study showed that CCT3 overexpression might affect the progression of multiple myeloma through the JAK/STAT3 pathway [18]. In addition, CCT was found to bind to STAT3 through its subunit CCT3, and therefore to affect the biosynthesis and activity of STAT3 [19]. Taken together, we propose that the JAK2/STAT3 pathway may be responsible for the CCT3-mediated cisplatin resistance in lung cancers. To verify this hypothesis, we performed *in vitro* and *in vivo* experiments in this study. We demonstrated that CCT3 could promote cisplatin resistance of lung adenocarcinoma (LUAD) cells through the JAK2/STAT3 pathway.

## Materials and methods

### Cell culture

Human LUAD A549 cells and cisplatin-resistant A549/DDP cells were purchased from the Cell Bank of the Chinese Academy of Sciences (Shanghai, China) and routinely tested to exclude the presence of mycoplasma contamination. A549 and A549/DDP cells were both cultured in Roswell Park Memorial Institute (RPMI)-1640 medium (Gibco, NY, USA) containing 10% fetal bovine serum (FBS, Gibco) and 1% penicillin/streptomycin (Beyotime, Shanghai, China). In order to maintain drug resistance, culture medium for A549/DDP cells was also supplemented with cisplatin (2 mg/L, Sigma, St Louis, USA) as previously reported [20]. All cells were cultured at 37°C in a 5% CO_2_ incubator.

### Gene expression profiling interactive analysis (GEPIA) dataset analysis

GEPIA is a web-portal (http://gepia.cancer-pku.cn/) for analyzing RNA sequencing data from the Cancer Genome Atlas (TCGA) and Genotype-Tissue Expression (GTEx) databases [21]. We utilized GEPIA to analyze the mRNA expression data of CCT3 in LUAD, lung squamous cell carcinoma (LUSC) and normal lung samples, and generate the survival curves.

### Cell viability assay

As previously described [22], cells were plated into a 96-well culture plate (2 × 10^3^ cells per well) and incubated with various concentrations of cisplatin (5, 10, 20, 40 μM) or sterile saline water (control, 0 μM) for 24 h. AG490 (Sigma, 20 μM) and IL-6 (Sigma, 50 ng/mL) were administrated to block and activate the JAK2/STAT3 pathway, respectively. According to the manufacturer’s instructions, cell viability was subsequently tested using the Cell Counting Kit-8 (Dojindo Laboratories, Kyushu, Japan). Absorbance values were measured at the wavelength of 450 nm using a microplate reader (Bio-Rad, Hercules, USA). Cell viability (%) = (ODcisplatin /ODcontrol) × 100%. The experiments were performed in triplicate.

### Quantitative reverse transcription polymerase chain reaction (RT-PCR)

Quantitative RT-PCR was performed as previously described, with some modifications [23]. Briefly, total RNA was extracted using the Trizol reagent (Beyotime) and immediately synthesized to cDNA using a PrimeScript™ RT reagent Kit (TaKaRa, Dalian, China). PCR primers were as follows: CCT3, forward: 5ʹ-CCTCCAGGTATCTTTTCCACTCT-3ʹ; reverse: 5ʹ-TCAGTCGGTGGTCATCTTTGG-3ʹ; Glyceraldehyde-3-phosphate dehydrogenase (GAPDH), forward: 5ʹ-GACAGTCAGCCGCATCTTCT-3ʹ; reverse: 5ʹ-TTAAAAGCAGCCCTGGTGAC-3ʹ. Quantitative RT-PCR was performed in a StepOnePlus™ Real-time PCR system (Life Technologies, Carlsbad, CA) with the SYBR Green Master Mix (TaKaRa). Thermocycling conditions were as follows: 94°C for 2 min, 94°C for 5 sec, 60°C for 15 sec, 72°C for 30 sec (40 cycles). The 2^−ΔΔCt^ method was used to calculate fold changes in the mRNA expression of CCT3 normalized to GAPDH.

### Western blot analysis

Western blot analysis was performed as previously described [24]. Briefly, total protein was isolated from cells using the RIPA lysis buffer (Beyotime). After measuring the protein concentration using a BCA kit (Beyotime), a total of 40 μg protein was separated by 8% or 15% sodium dodecyl sulfate-polyacrylamide gel electrophoresis gels and transferred onto the polyvinylidene fluoride membranes (Merck Millipore, Darmstadt, Germany). Membranes were subsequently blocked with 5% skim milk at room temperature for 2 h, and incubated at 4°C overnight with primary antibodies: anti-CCT3 (Abcam, Cambridge, UK, ab225878, 1:2,000), anti-cyclin B1 (Abcam, ab181593, 1:2,000), anti-cyclin dependent kinase 1 (CDK1, Abcam, ab133327, 1:10,000), anti-Cleaved Caspase-3 (Abcam, ab2302, 1:500), anti-STAT3 (Abcam, ab119352, 1:5,000), anti-p-STAT3 (Abcam, ab76315, 1:5,000), anti-JAK2 (Cell Signaling Technology, Inc., MA, USA, #3230, 1:1,000), anti-p-JAK2 (Cell Signaling Technology, #4406, 1:1,000), and anti-GAPDH (Beyotime, AG019, 1:1,000). After incubation with corresponding horseradish peroxidase-conjugated secondary antibodies (Beyotime, A0208, A0216, 1:5,000) at room temperature for 2 h, membranes were visualized under a ChemiDoc XRS+ system (Bio-Rad). The protein bands were quantified using the Image J software (National Institutes of Health, Bethesda, USA).

### RNA interference

The pGLV/H1/GFP-lentivirus vector containing the shRNA sequence targeting to CCT3 (shCCT3) and negative control vector (shNC) were purchased from GenePharma (Shanghai, China). The target sequence of shCCT3 was as follow: 5ʹ-CAAGTCCATGATCGAAATT-3ʹ. Cell transfection was conducted as previously described, with some modifications [25]. Briefly, A549/DDP cells were infected with recombinant lentivirus at a multiplicity of infection of 50. Stable cell lines expressing shCCT3 or shNC were acquired by screening with 0.5 μg/mL puromycin. Interference efficiency was tested using quantitative RT-PCR and western blot analysis.

### Wound healing assay

Wound healing assay was performed as previously described [26]. Briefly, the transfected A549/DDP cells were plated into a 6-well culture plate and cultured until 95% confluence. A linear wound was then created in the cell monolayer using a sterile 10-µl pipette tip. After being washed twice with sterile phosphate buffer saline (PBS), cells were cultured in FBS-free RPMI-1640 medium dissolved with cisplatin (20 μM) at 37°C for 24 h. Wounds were observed and photographed at 0 h and 24 h under an inverted fluorescence microscope (Olympus, Tokyo, Japan). The experiments were performed in triplicate.

### Transwell assay

Transwell assay was performed with the 24-well transwell chambers with 8 μm pores (Millipore, Billerica, USA) as previously described, with some modifications [27]. Briefly, before seeding cells, the upper chamber was added with 100 μL matrigel (BD, USA) diluted in RPMI-1640 medium at the ratio of 1:8 and maintained at 37°C for 6 h. Then, the transfected A549/DDP cells were plated into the upper chamber at a density of 1 × 10^5^ and cultured in FBS-free RPMI-1640 medium dissolved with cisplatin (20 μM). RPMI-1640 medium containing 10% FBS was added into the lower chamber. After being cultured at 37°C for 48 h, the invaded cells on the lower surface of the membrane were fixed with 4% paraformaldehyde (Boster, Wuhan, China) for 30 min, stained with 0.1% crystal violet (Beyotime) for 15 min and then washed thrice with PBS. Finally, the invaded cells were viewed and photographed using an inverted optical microscope (Olympus). The experiments were performed in triplicate.

### Flow cytometry analysis

Flow cytometry analysis was conducted as previously described [28]. For cell cycle analysis, the transfected A549/DDP cells were incubated with cisplatin (20 μM) at 37°C for 24 h. Subsequently, cells were harvested and fixed in 70% ethanol overnight at 4°C, resuspended with PBS, and stained with 50 μg/mL propidium iodide (PI, Sigma) in the dark for 30 min. For apoptosis analysis, the transfected A549/DDP cells were treated with cisplatin (20 μM) or co-treated with cisplatin and AG490 (20 μM) or IL-6 (50 ng/mL) at 37°C for 24 h. Then, cells were collected and washed twice with ice-cold PBS, and stained with 5 μL Annexin-V-fluorescein isothiocyanate (FITC)/PI (Sigma) in the dark at room temperature for 15 min. Stained cells were ultimately analyzed using a FACScan flow cytometry (Becton Dickinson, San Jose, CA). The experiments were performed in triplicate.

### Colony formation assay

Colony formation assay was performed as previously described [29]. Briefly, the transfected A549/DDP cells were seeded into a 6-well culture plate at a density of 500 cells per well and incubated with cisplatin (Sigma, 20 μM) for 2 weeks. The colonies were then fixed with 4% paraformaldehyde (Boster) for 30 min, stained with 0.1% crystal violet (Beyotime) for 15 min and washed twice with PBS. The colonies were subsequently photographed and counted. The experiments were performed in triplicate.

### Xenograft tumor assay

Twelve male nude mice (4-week-old) were purchased from Laboratory Animal Resource, Chinese Academy of Sciences (Shanghai, China), and randomly divided into the shNC and shCCT3 groups (n = 6). Xenograft tumor assay was performed as previously described, with some modifications [30]. Briefly, the transfected A549/DDP cells (5 × 10^6^) were subcutaneously injected into the right flank of each nude mouse. After injection for 1 week, all nude mice were intraperitoneally administered with cisplatin (5 mg/kg) once a week for 3 weeks. Tumor diameters were measured every week and the tumor volume was calculated as the following equation: volume (mm^3^) = (shortest diameter)^2^ × (longest diameter)/2. After injecting cells for 4 weeks, all nude mice were euthanized and xenograft tumors were collected and weighed. All animal experiments were conducted in accordance with the National Institutes of Health guide for the care and use of laboratory animals, and were approved by the Institutional Animal Care and Use Committee of the First Affiliated Hospital of Hainan Medical College.

### Statistical analysis

Data are presented as the mean ± S.D. Statistical analyses were performed with SPSS for windows, version 22.0 (SPSS Inc., Chicago, IL). Unpaired 2-tailed Student’s t test or one-way ANOVA was used to calculate the significant difference between each group. A p-value < 0.05 was considered to indicate a statistically significant difference.

## Results

Although CCT3 has been found to be closely related to the tumorigenesis and progression of various cancers [9–11], its potential role in drug resistance of lung cancer and the underlying mechanisms remain unclear. The present study aimed to investigate a) whether CCT3 can affect the cisplatin resistance of LUAD cells, and b) whether the JAK2/STAT3 pathway is involved in this process, using both *in vitro* and *in vivo* models. We first analyzed CCT3 expression in NSCLC tissues, human LUAD A549 cells, and the cisplatin-resistant A549/DDP cells, and found that CCT3 was upregulated. Next, we performed *in vitro* and *in vivo* assays and discovered that CCT3 knockdown re-sensitized the drug-resistant A549/DDP cells to cisplatin treatment. Finally, we bidirectionally intervened the JAK2/STAT3 pathway and found that this pathway might contribute to the CCT3-mediated cisplatin resistance in LUAD cells.

### CCT3 was overexpressed in NSCLCs and correlated with poor prognosis in LUAD patients

**Figure 1.**
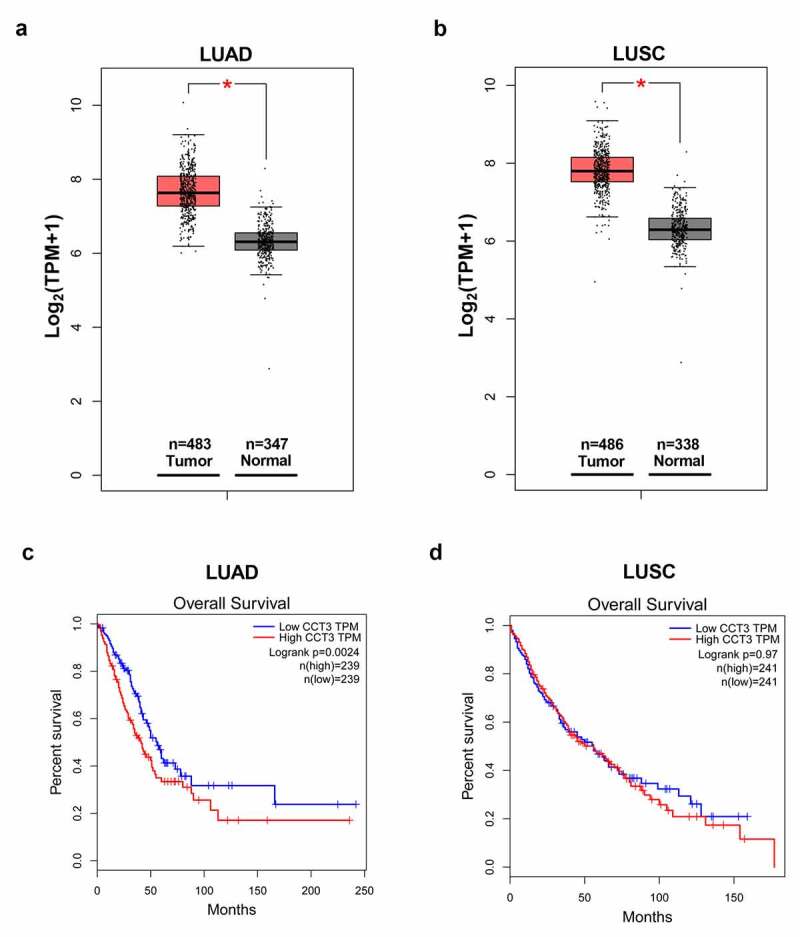
CCT3 was overexpressed in NSCLCs and correlated with poor prognosis in LUAD patients. (a, b) Analyses of CCT3 mRNA expression data in LUAD, LUSC and normal lung samples using the GEPIA web-portal. (c, d) Overall survival of LUAD and LUSC patients with low or high CCT3 expression using the GEPIA web-portal. *P < 0.05 between tumor samples and normal samples

We first surveyed the expression of CCT3 in NSCLC tissues from the TCGA and GTEx projects using the GEPIA web server. The results demonstrated that CCT3 expression was significantly upregulated in both LUAD and LUSC tissues compared to that in normal lung tissues (Figure 1(a,b)). Furthermore, as shown in Figure 1(c), LUAD patients with low CCT3 expression showed significantly better overall survival rate than those with high CCT3 expression. On the contrary, overall survival rate between LUSC patients with low and high CCT3 expression levels showed no significant difference (Figure 1(d)).

### CCT3 expression was significantly up-regulated in A549/DDP cells

**Figure 2.**
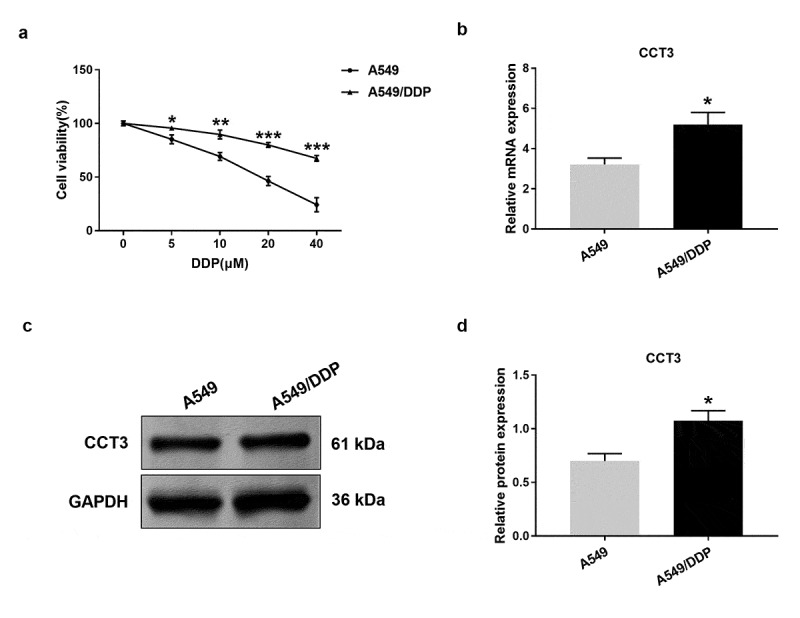
CCT3 expression was significantly up-regulated in A549/DDP cells. (a) Cell viability assay of A549/DDP and A549 cells upon cisplatin (0–40 μM) treatments. *P < 0.05, **P < 0.01 and ***P < 0.001 between A549/DDP cells and A549 cells. (b) Relative CCT3 mRNA expression level in A549 and A549/DDP cells normalized to GAPDH. (c) Western blot analysis of CCT3 in A549 and A549/DDP cells. (d) Relative CCT3 protein level normalized to GAPDH. *P < 0.05 between A549/DDP cells and A549 cells

We next examined CCT3 expression across multiple cell lines. We found that upon the administration of gradient concentrations of cisplatin (5–40 μM), A549/DDP cells exhibited significantly smaller reduction in cell viability than A549 cells at each concentration (Figure 2(a)). These results confirmed the robustness of our assays and the cisplatin-resistant phenotype of A549/DDP cells. Furthermore, the results showed that both mRNA and protein expression levels of CCT3 in A549/DDP cells were significantly higher than those in A549 cells (Figure 2(b–d)).

### CCT3 knockdown significantly suppressed the proliferation, migration and invasion in the cisplatin-treated A549/DDP cells

**Figure 3.**
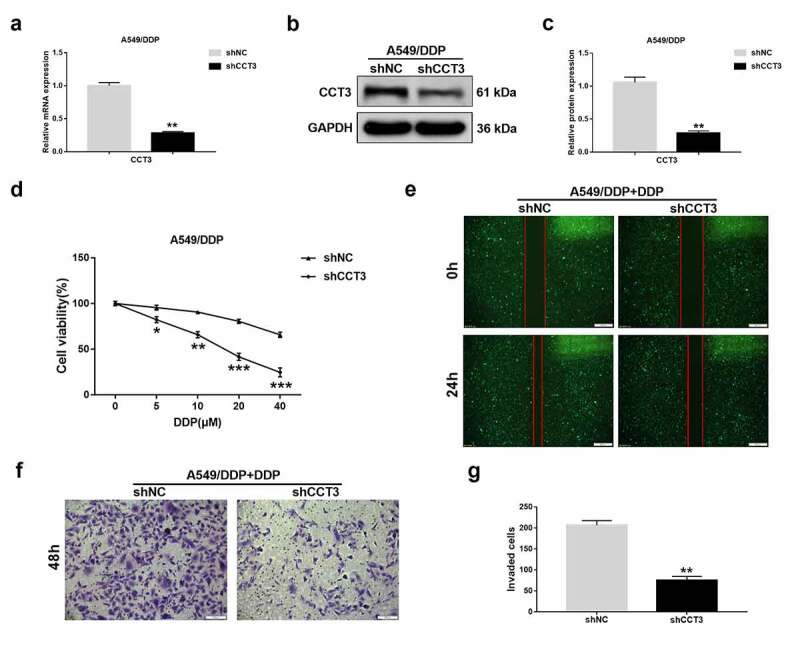
CCT3 knockdown significantly suppressed the proliferation, migration and invasion in cisplatin-treated A549/DDP cells. (a) Relative CCT3 mRNA expression level in A549/DDP cells normalized to GAPDH after transfection with lentiviral shRNA targeting to CCT3 (shCCT3) and negative control vector (shNC). (b) Western blot analysis of CCT3 in shRNA-treated A549/DDP cells. (c) Relative CCT3 protein level normalized to GAPDH. (d) Cell viability assay of shRNA-treated A549/DDP cells upon cisplatin (0–40 μM) treatments. (e) Wound healing assay of shRNA-treated A549/DDP cells upon cisplatin (20 μM) treatment; scale bar: 500 μm. (f) Transwell assay of shRNA-treated A549/DDP cells upon cisplatin (20 μM) treatment; scale bar: 100 μm. (g) Statistics of invaded cells. *P < 0.05, **P < 0.01 and ***P < 0.001 between shCCT3 group and shNC group

To determine the effects of CCT3 manipulation in the cisplatin-resistant A549/DDP cells, we stably knocked down CCT3 expression in A549/DDP cells using lentiviral shRNA. Compared to those in the negative control, both mRNA and protein levels of CCT3 were significantly decreased in A549/DDP cells upon shCCT3 treatment (Figure 3(a–c)). Remarkably, when CCT3 was knocked down, the cisplatin-induced cell viability reduction of A549/DDP cells was significantly enhanced (Figure 3(d)). To further determine the cellular effects of CCT3 knockdown on cisplatin resistance, we focused on A549/DDP cells treated with 20 μM cisplatin in the following experiments as an example. Consistent with the cell viability results, A549/DDP cells treated with shCCT3 showed notably weaker migration capacity than that treated with the control shRNA (Figure 3(e)). In addition, compared to the control, the cisplatin-treated A549/DDP cells were less invasive upon CCT3 knockdown (Figure 3(f,g)). These results suggested that CCT3 knockdown could modulate several cellular phenotypes of cisplatin-treated A549/DDP cells that are normally resistant to drug treatment.

### CCT3 knockdown induced G2/M phase arrest and facilitated apoptosis in the cisplatin-treated A549/DDP cells

**Figure 4.**
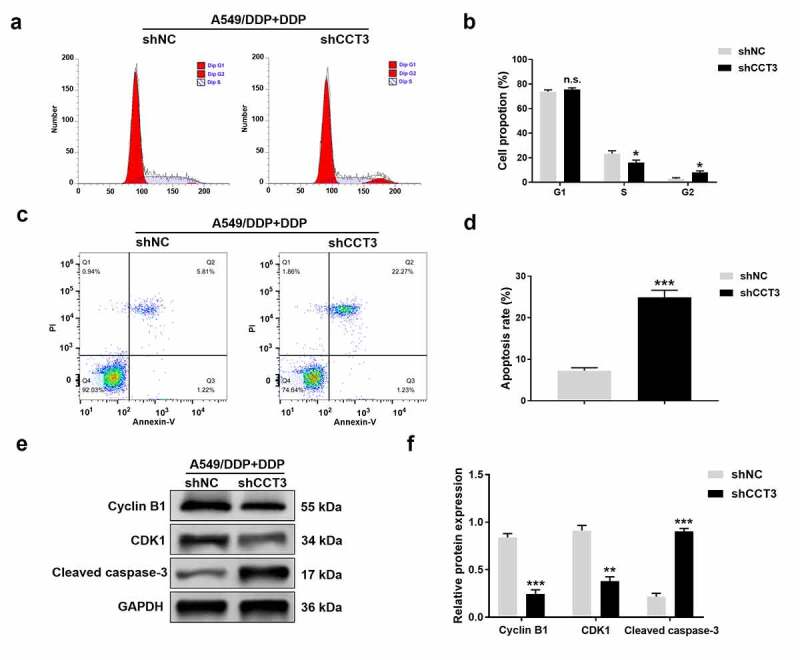
CCT3 knockdown induced G2/M phase arrest and facilitated apoptosis in cisplatin-treated A549/DDP cells. (a) Cell cycle analysis of shRNA-treated A549/DDP cells upon cisplatin (20 μM) treatment. (b) Statistical analysis of the cell cycle distribution. (c) Apoptosis analysis in shRNA-treated A549/DDP cells upon cisplatin (20 μM) treatment. (d) Statistical analysis of apoptosis rate. (e) Western blot analysis of cyclin B1, CDK1 and cleaved caspase-3 in shRNA-treated A549/DDP cells upon cisplatin (20 μM) treatment. (f) Relative protein expression levels of cyclin B1, CDK1 and cleaved caspase-3 normalized to GAPDH. *P < 0.05, **P < 0.01, ***P < 0.001 and n.s., no significance, between shCCT3 group and shNC group

We next examined how CCT3 knockdown affected cell cycle progression and apoptosis. Flow cytometry analyses revealed that upon the administration of 20 μM cisplatin, CCT3 knockdown significantly decreased the percentage of A549/DDP cells in the S phase while increasing the G2 phase population. However, CCT3 knockdown did not modulate the proportion of A549/DDP cells in the G1 phase (Figure 4(a,b)). These findings suggested that knockdown of CCT3 expression allowed cisplatin to arrest A549/DDP cells in G2/M phase.

We also found that the proportion of apoptotic A549/DDP cells following the cisplatin treatment was significantly increased when CCT3 was knocked down (Figure 4(c,d)). In addition, the shCCT3 group had significantly reduced CDK1 and cyclin B1 at protein levels, and elevated the protein expression of cleaved caspase-3 in cisplatin-treated A549/DDP cells, compared to those in the shNC group (Figure 4(e,f)).

### CCT3 ablation weakened the tumorigenicity of cisplatin-treated A549/DDP cells in vitro and in vivo

**Figure 5.**
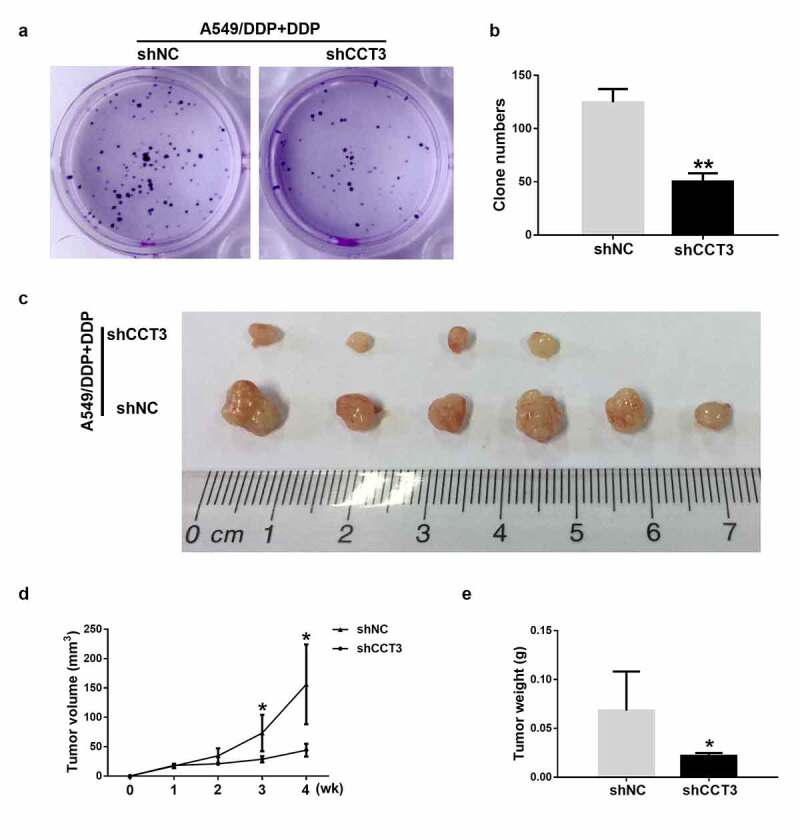
CCT3 ablation weakened the tumorigenicity of cisplatin-treated A549/DDP cells *in vitro* and *in vivo*. (a) Colony formation assay of shRNA-treated A549/DDP cells upon cisplatin (20 μM) treatment. (b) Statistics of the clone number. (c) Xenograft tumor assay of shRNA-treated A549/DDP cells in nude mice upon cisplatin (5 mg/kg) treatment once a week for 3 weeks. (d, e) Statistical analysis of tumor volume and tumor weight. *P < 0.05 and **P < 0.01 between shCCT3 group and shNC group

CCT3 knockdown also affected the colony formation capacity of the cisplatin-treated A549/DDP cells. Upon the treatment of 20 μM cisplatin, the quantity of colonies formed by A549/DDP cells was significantly reduced when CCT3 was simultaneously knocked down (Figure 5(a,b)). More importantly, upon the treatment of cisplatin (5 mg/kg), the tumor formation rate of the shCCT3-treated A549/DDP cells in nude mice was lower than the shNC-treated cells (4/6 in shCCT3 group vs 6/6 in shNC group, Figure 5(c)). The volume and weight of xenograft tumors were also significantly smaller in the shCCT3 group compared to those in the shNC group (Figure 5(d,e)). The aforementioned results suggested that knockdown of CCT3 expression by the shCCT3 significantly weakened the tumorigenicity of cisplatin-treated A549/DDP cells *in vitro* and *in vivo*.

### CCT3 knockdown induced re-sensitization of A549/DDP cells to cisplatin via inhibiting the JAK2/STAT3 pathway

**Figure 6.**
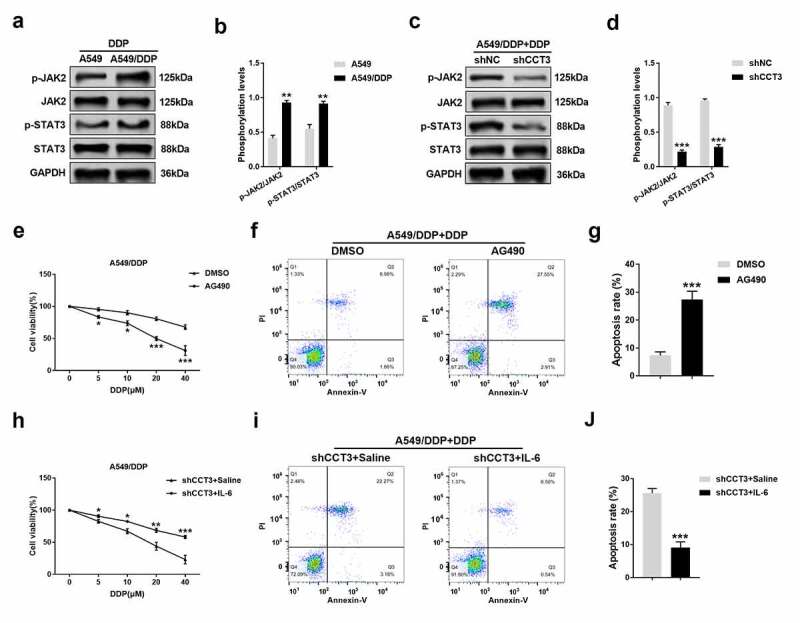
CCT3 knockdown induced re-sensitization of A549/DDP cells to cisplatin via suppressing blocking the JAK2/STAT3 pathway. (a, c) Upon cisplatin (20 μM) treatment, western blot analysis of p-JAK2, JAK2, p-STAT3 and STAT3 in A549/DDP and A549 cells or in shRNA-treated A549/DDP cells. (b, d) Statistical analysis of phosphorylation levels of JAK2 and STAT3. **P < 0.01 between A549/DDP cells and A549 cells; ***P < 0.001 between shCCT3 group and shNC group. (e) Cell viability assay of A549/DDP cells upon treatments of cisplatin (0–40 μM) with AG490 (20 μM) or DMSO. *P < 0.05 and ***P < 0.001 between AG490 and DMSO. (f) Apoptosis analysis in A549/DDP cells after administration of cisplatin (20 μM) with AG490 (20 μM) or DMSO. (g) Statistical analysis of apoptosis rate. ***P < 0.001 between AG490 and DMSO. (h) Cell viability assay of shCCT3-treated A549/DDP cells upon treatments of cisplatin (0–40 μM) with IL-6 (50 ng/mL) or saline water. *P < 0.05, **P < 0.01 and ***P < 0.001 between shCCT3+ IL-6 group and shCCT3+ saline group. (i) Apoptosis analysis in shCCT3-treated A549/DDP cells after administration of cisplatin (20 μM) with IL-6 (50 ng/mL) or saline water. (j) Statistical analysis of apoptosis rate. ***P < 0.001 between shCCT3+ IL-6 group and shCCT3+ saline group

Finally, whether the JAK2/STAT3 pathway is involved in the regulation of cisplatin resistance by CCT3 was investigated. Upon the treatment of 20 μM cisplatin, the phosphorylation levels of JAK2 and STAT3 were significantly enhanced in A549/DDP cells compared to those in A549 cells (Figure 6(a,b)). Remarkably, the enhanced phosphorylation was abolished when CCT3 was knocked down in A549/DDP cells (Figure 6(c,d)). After blocking the JAK2/STAT3 pathway by 20 μM AG490, the toxic effects of gradient concentrations of cisplatin on A549/DDP cells were significantly strengthened (Figure 6(e)), and the apoptotic rate of A549/DDP cells induced by 20 μM cisplatin was significantly enhanced (Figure 6(f,g)). Moreover, following the activation of the JAK2/STAT3 pathway by 50 ng/mL IL-6, the toxic effects of gradient concentrations of cisplatin on shCCT3-treated A549/DDP cells were significantly weakened (Figure 6(h)), and the shCCT3-induced increase in A549/DDP cell apoptosis upon the treatment of 20 μM cisplatin was also significantly alleviated (Figure 6(i,j)).

## Discussion

CCT3 has been shown to be overexpressed in breast and liver cancers, and its expression level is inversely correlated with the patient survival rate [11,31]. In papillary thyroid carcinoma (PTC), CCT3 was also upregulated and its knockdown significantly suppressed the proliferation of PTC cells [32]. Furthermore, it has been reported that increased expression level of CCT3 predicted the progression and adverse outcomes of multiple myeloma patients [18]. Consistently, our survey using the GEPIA web-portal also demonstrated that CCT3 expression level was significantly upregulated in both LUAD and LUSC tissues. Furthermore, our results revealed that high expression level of CCT3 was associated with the poor prognosis in LUAD patients, albeit not in LUSC patients. Therefore, we hypothesized that CCT3 may be closely involved in the tumorigenesis and progression of NSCLCs, and it might have a more prominent role in LUAD, which would benefit from further studies.

Although CCT3 has been widely investigated in diverse cancers, its involvement in drug resistance remains elusive. In this study, we attempted to explore the potential role of CCT3 in regulating cisplatin resistance in LUAD cells utilizing the well-established cisplatin-resistant derivative of A549 cells, A549/DDP cells. A previous work revealed that the transcription of another CCT subunit, CCT5, was significantly increased in p53-mutated breast cancers, which might underlie the low response rate of such cancer cells to docetaxel [33]. Furthermore, it was reported that knockdown of CCT8 expression enhanced the inhibitory effects of cisplatin on esophageal squamous cell carcinoma TE-1 cells [34]. These findings suggest that the subunits of CCT could be responsible for the drug resistance in cancer cells. Additionally, a proteomic analysis has revealed that CCT2 and CCT3 levels were increased in adriamycin-resistant squamous lung cancer DLKP cells [35]. Consistently, our results demonstrated that CCT3 expression was significantly upregulated in A549/DDP cells. This result indicated a potential role of CCT3 in regulating cisplatin resistance of LUAD cells.

To assess the functional consequence of CCT3 in cisplatin resistance, we successfully knocked down CCT3 using a lentiviral shRNA and examined various cellular phenotypes. The A549/DDP cells exposed to cisplatin and shCCT3 showed not only reduced proliferation but also impeded migration/invasion capability. Furthermore, CCT3 knockdown notably arrested the cell cycle of cisplatin-treated A549/DDP cells in G2/M phase. Cyclin B1 is a well-established G2 phase-associated cyclin that can bind to and activate CDK1, which is an indispensable step for cells entering into mitosis [36]. As expected, we found that cyclin B1 and CDK1 protein levels in cisplatin-treated A549/DDP cells were significantly reduced after CCT3 knockdown. Here, we propose that the increased CCT3 expression may contribute to the uncontrolled proliferation of cisplatin-treated A549/DDP cells, possibly by promoting cyclin B1/CDK1 expression and consequently accelerated cell cycle progression. It is worth noting, however, that silencing CCT3 could also cause S phase arrest in breast cancer cells [25] and hepatocellular carcinoma cells without any additional treatments [37]. These and our findings suggest that CCT3 may exert different effects on cell cycle in different tumor contexts that bear distinct endogenous and exogenous stimuli to start with. In addition, our results revealed that CCT3 knockdown significantly promoted apoptosis in cisplatin-treated A549/DDP cells. This was exemplified by an increased apoptosis rate and enhanced expression of an apoptotic marker, cleaved caspase-3 [38]. More significantly, our *in vitro* and *in vivo* results both demonstrated that CCT3 knockdown prominently weakened the tumorigenicity of A549/DDP cells upon cisplatin treatment. Taken together, the aforementioned results revealed the functional significance of CCT3 in promoting the cisplatin resistance of LUAD cells, and indicated that CCT3 knockdown can re-sensitize A549/DDP cells to cisplatin.

Previous work has identified the JAK2/STAT3 pathway to be involved in cisplatin resistance of nasopharyngeal carcinoma [16] and epirubicin resistance of NSCLCs [17]. It was also reported that the phosphorylation levels of JAK2 and STAT3 were significantly elevated in cisplatin-resistant NSCLC cell lines compared with those in cisplatin-sensitive NSCLC cell lines [39]. These findings indicate a plausible link between the JAK2/STAT3 pathway and cisplatin resistance of NSCLCs. Furthermore, several studies have revealed the relationship between CCT3 and the JAK/STAT3 pathway. First, bioinformatic analyses of multiple myeloma identified CCT3 targeted genes that were involved in the JAK/STAT3 pathway [18]. Second, CCT3 could bind to STAT3 and affect its biosynthesis and activity [19]. Finally, CCT3 knockdown could reduce both total and phosphorylated STAT3 levels in hepatocellular carcinoma HepG2 cells [37]. We therefore hypothesized that the JAK2/STAT3 pathway could be regulated by CCT3 and might contribute to cisplatin resistance in LUAD cells. Consistent with the previous studies [37,39], our study also demonstrated that the phosphorylation levels of JAK2 and STAT3 in A549/DDP cells were significantly higher than those in A549 cells, and their phosphorylation levels were significantly reduced by CCT3 knockdown. Furthermore, we subsequently blocked and activated the JAK2/STAT3 pathway using AG490 and IL-6, respectively, as previously described [40,41]. Our results demonstrated that AG490 treatment mimicked the inhibitory effects of CCT3 knockdown on A549/DDP cells upon cisplatin treatment while IL-6 rescued the cisplatin resistance of shCCT3-treated A549/DDP cells. Taken together, we suggest that CCT3 could promote cisplatin resistance of LUAD cells by regulating the JAK2/STAT3 pathway.

Our present study reveals a detailed mechanism by which CCT3 and JAK2/STAT3 pathway contribute to cisplatin resistance of LUAD cells. This provides additional insights into valuable targets for future clinical interventions. However, a limitation of our present study is that we just conducted loss-of-function assays of CCT3 in A549/DDP cells using the lentiviral-based gene silencing. Gain-of-function assays based on the overexpression of CCT3 in A549 cells upon the cisplatin treatment should also be performed in our further studies.

## Conclusion

In conclusion, our study revealed that CCT3 could activate the JAK2/STAT3 pathway to facilitate cisplatin resistance of LUAD cells. Our results indicate that CCT3 could serve as a novel molecular target for overcoming cisplatin resistance in LUAD patients.

## Data Availability

The data that support the findings of this study are available from the corresponding author upon reasonable request.
